# Challenges in the diagnosis and management of isolated congenital complete atrioventricular block in premature newborns

**DOI:** 10.1002/ccr3.2190

**Published:** 2019-05-09

**Authors:** Rūta Gegieckienė, Ramunė Vankevičienė, Odeta Kinčinienė, Arūnas Liubšys

**Affiliations:** ^1^ Vilnius University, Faculty of Medicine Institute of Clinical Medicine, Clinic of Children's Diseases Vilnius Lithuania

**Keywords:** dilated cardiomyopathy, fetal bradycardia, isolated congenital complete atrioventricular block, pacemaker implantation, premature newborns

## Abstract

Diagnostic and treatment challenges of congenital complete atrioventricular block in two premature newborn babies are presented. Timely recognition of this fetal condition, appropriate antenatal care, and treatment at a tertiary level care hospital as well as prompt postnatal management of the newborn baby are the key factors for good outcome. Prematurity is also associated with an additional risk of poor outcome and complications.

## 1 INTRODUCTION

Neonates affected by isolated congenital complete atrioventricular block (isolated CCAVB) are at risk of heart failure or sudden cardiac death. The management of isolated CCAVB in premature newborns is challenging. This manuscript reports diagnostic and treatment challenges of isolated CCAVB in two low birth weight premature neonates (1060 and 1700 g) and different outcomes.

Isolated CCAVB is a rare and serious condition, which is caused by transplacentally acquired maternal antibodies. The incidence is one in 15 000/20 000 live births.^1^, ^2^ Approximately 15%‐30% of infants with CCAVB die in utero or the first months of life due to severe cardiac damage and various cardiovascular complications.^3^ A number of associated diseases and conditions (eg, fetal hydrops, fetal bradycardia—<55 beats per minute—ventricular dysfunction, low birth weight, prematurity, and structural heart disease) can complicate CCAVB and increase mortality.^4^ According to different authors, up to 65% of neonates, infants, and children with this condition require pacemaker implantation as a life‐saving procedure.^3^

It is important to distinguish fetal CCAVB from other obstetric causes of fetal bradycardia. Mistakenly assuming fetal bradycardia as a sign of acute fetal distress due to fetal hypoxia, but not due to CCAVB can lead to unnecessary emergency cesarean section, but not to appropriate antenatal treatment of CCAVB and prolongation of pregnancy.

Pacemaker implantation techniques and pacemakers have undergone significant improvements recently and are now available for low birth weight preterm neonates. Nevertheless, prematurity, low birth weight, hemodynamic instability, and metabolic acidosis all independently complicate successful pacemaker implantation, as well as the ongoing management and outcomes.^3^ There are a few reports of treatment options in the management of preterm neonates affected by isolated CCAVB.^2^, ^4^, ^5^, ^6^, ^7^, ^8^, ^9^, ^10^ In this report, we present our first experience with the management of isolated CCAVB in two low birth weight preterm newborns and discuss the potential reasons for the different outcomes.

## 2 CASE PRESENTATIONS

### 2.1 Case 1

**Figure 1:**
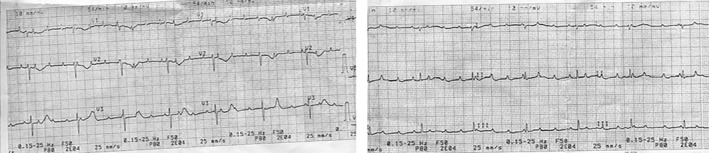
Case 1. ECG after birth—isolated congenital complete atrioventricular block

A 32‐year‐old mother, diagnosed with undifferentiated connective tissue disease and positive autoimmune antibodies (anti‐ds‐DNA, ANA, anti‐SSB, and anti‐SSA), was admitted to a regional hospital with suspected fetal hypoxia at the 29th week of gestation. The fetal heart rate was 52 beats per minute. Subsequently, due to suspected fetal hypoxia, an emergency cesarean section was performed, producing a 1700‐g‐male neonate with Apgar scores of 4 at 1 minute and 7 at 5 minutes. The baby had signs of fetal hydrops and cardiopulmonary insufficiency, bradycardia (52 beats per minute), poor peripheral perfusion, hypotonia, and hyporeflexia. Due to elevated proinflammatory markers (C‐reactive protein, procalcitonin) and poor baby condition, antibiotics were prescribed for suspected early onset of neonatal infection. Endotracheal intubation, surfactant (Curosurf®) instillation, and mechanical lung ventilation were started in the delivery room because of respiratory distress, and hemodynamic stability was maintained with a continuous infusion of dopamine. Electrocardiography demonstrated complete atrioventricular block (Figure 1), and the newborn was transferred to a tertiary medical center. Other echocardiographic features included an ejection fraction of 20%, dilated right and left heart chambers, patent foramen ovale, and an open ductus arteriosus.

**Figure 2:**
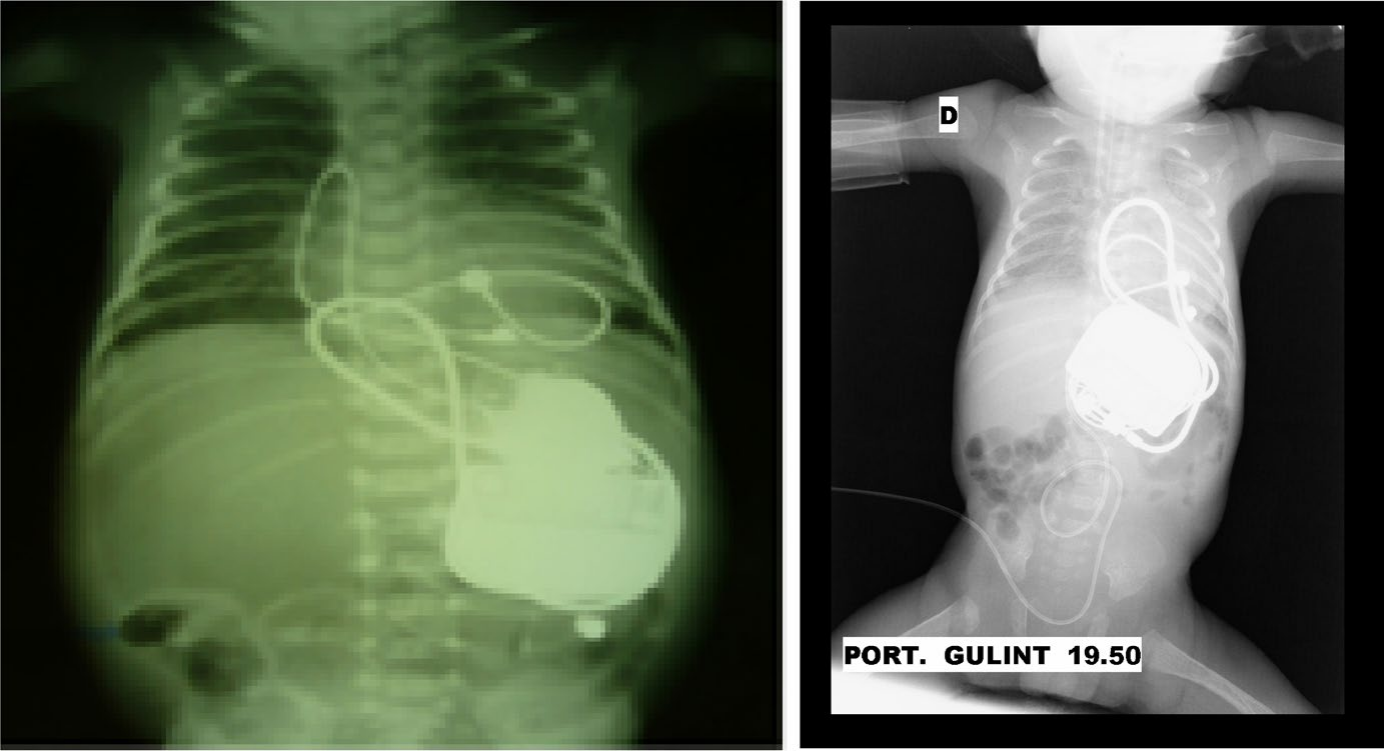
Chest X‐ray after epicardial pacemaker implantation (left—Case 1; right—Case 2)

**Figure 3:**
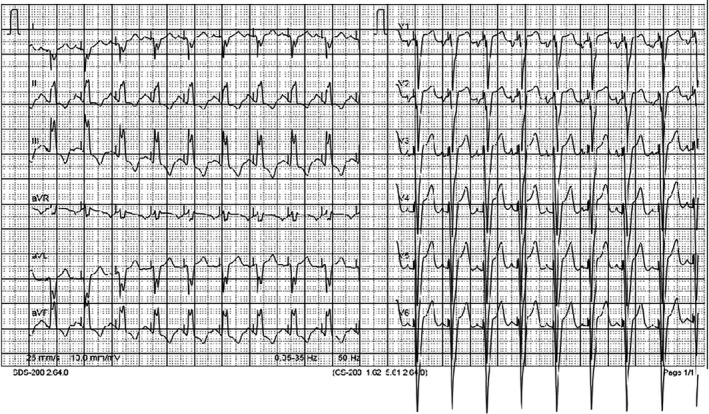
Case 1. ECG after pacemaker implantation. Left bundle branch block morphology and rightward QRS axis, which indicate pacing site from right ventricular outflow tract

On the second day of life, an epicardial pacemaker was implanted. The pericardium was opened under the xiphoid process through a small incision on the left epigastrium. A generator (Microny®—2525 T; St. Jude Medical, Inc) was placed under the rectus muscle and epicardial Medtronic leads were attached to the right ventricle (Figure 2). After pacemaker implantation, the electrocardiogram revealed a left bundle branch block morphology and rightward QRS axis (Figure 3). The pacemaker rate was programmed at 130 beats per minute.

Serial echocardiograms the following day revealed an increase in the left ventricular ejection fraction to 30%‐40% and second‐grade tricuspid regurgitation. Gradual improvement of the baby's general condition was noted over the next few days, but the course of the disease was later complicated by progressive left ventricular dilatation with systolic dysfunction and prematurity‐related complications. On the 10th day after the pacemaker insertion, a right‐sided pneumothorax requiring pleural drainage was diagnosed, and 2 days after that the clinical condition was complicated by necrotizing enterocolitis with intestinal perforation and peritonitis. The baby recovered from the intestinal complications after open abdominal surgery with resection of an ileal segment and direct bowel anastomosis. However, other complications included retinopathy of prematurity (stage 2), sensorineural hearing impairment, hypoxic‐ischemic encephalopathy with periventricular leukomalacia, anemia, and bilateral inguinal hernias.

The baby was discharged from the hospital at 13 weeks of age (42nd week of corrected age) with persistent congestive heart failure. The patient underwent anti‐heart failure treatment such as digoxin, diuretics (furosemide and spironolactone), ACE inhibitors (captopril), and beta‐blockers (carvedilol). Despite this, progressive left ventricular myocardial weakness was revealed on serial echocardiographic examinations. A follow‐up at 4 months of age showed significant heart chamber dilatation (the left ventricle diastolic diameter was 4.3 cm) and severe left ventricle failure with the left ventricle ejection fraction 18%. At 1 year of age, the child was added to the heart transplant recipient list, but because of progressive heart failure, the child died at 1.5 years of age.

### 2.2 Case 2

Significant fetal bradycardia was detected, and complete heart block was suspected at the 26th week of gestation on prenatal examination of a 33‐year‐old mother with autoimmune thyroiditis. Before this visit, the mother had a normal course of pregnancy without any suspicion of fetal distress. Emergency hospitalization to a tertiary level perinatal center was recommended. Autoimmune antibodies (anti‐SS‐A, anti‐Ro 52, and ANA) were identified in the blood of the pregnant woman, and antenatal steroids were given for fetal lung maturation. Two weeks later an emergency cesarean section was performed because of severe preeclampsia.

**Figure 4:**
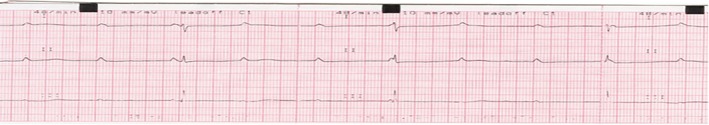
Case 2. ECG after birth—isolated congenital complete atrioventricular block

A 1060‐g‐female neonate with respiratory distress syndrome and bradycardia (40‐50 beats per minute) was delivered with Apgar scores of 7 at 1 minute and 8 at 5 minutes. The baby was transferred to the neonatal intensive care unit, where mechanical lung ventilation was started. The diagnosis of a complete atrioventricular block with a heart rate of 48 beats per minute was confirmed by electrocardiography (Figure 4). Echocardiography revealed a patent foramen ovale and open ductus arteriosus.

**Figure 5:**
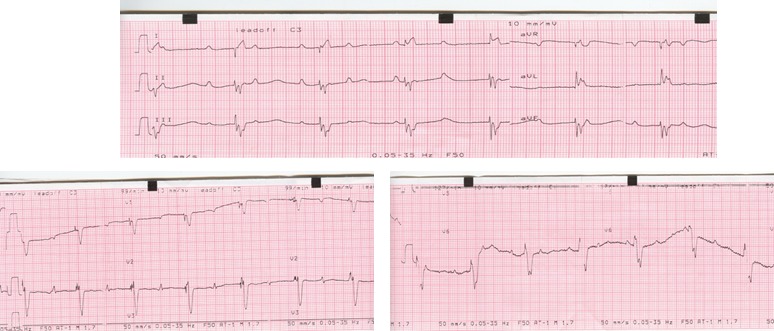
Case 2. ECG after pacemaker implantation

**Figure 6:**
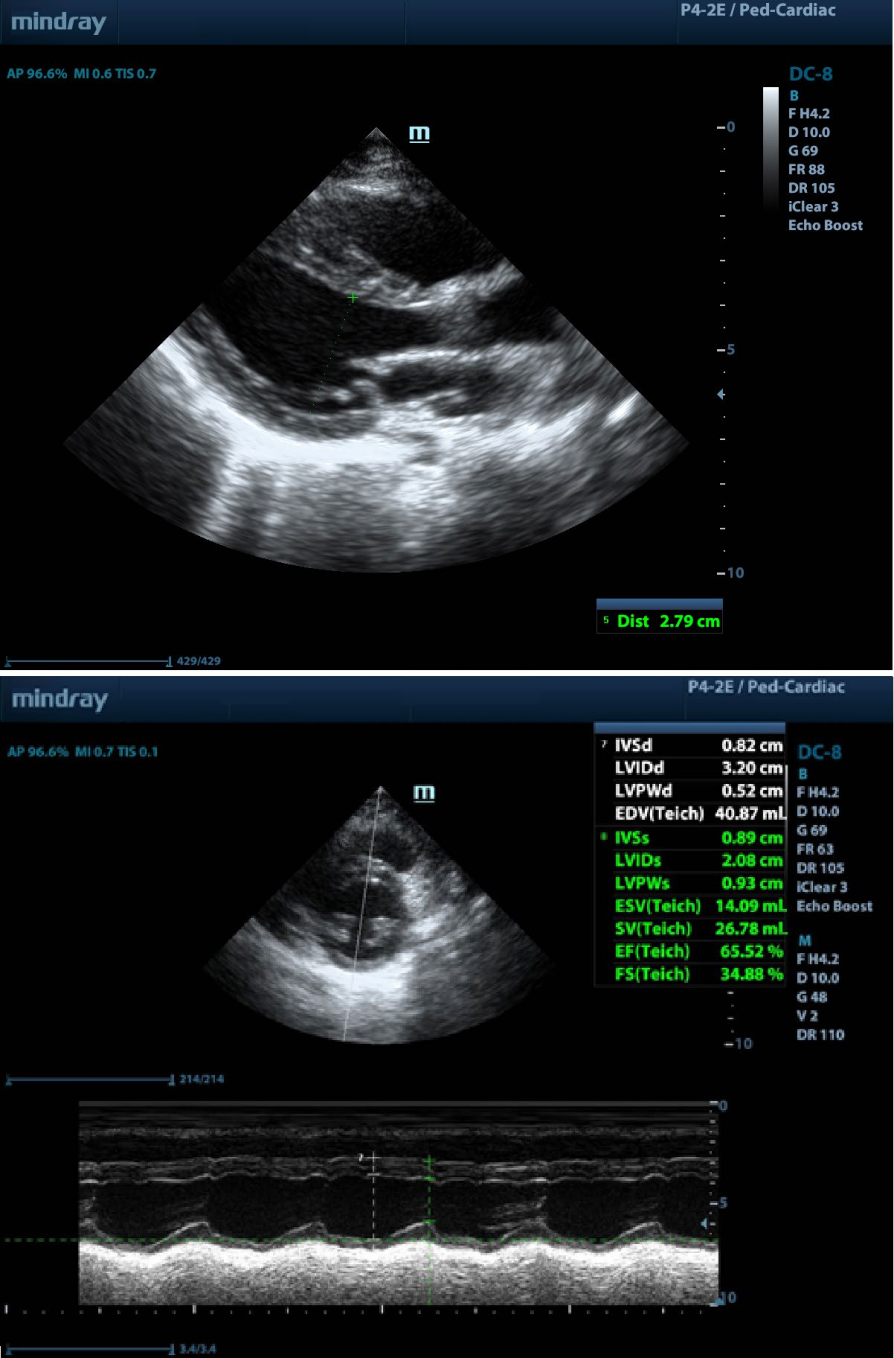
Case 2. The echocardiogram at 5 y of age with no heart chamber dilatation

Due to the rapid deterioration of the neonate's clinical condition, she underwent urgent pacemaker implantation at 4 hours of age. The pericardium was opened through a small epigastric incision, and a Medtronic steroid‐eluting leads were attached to the epicardium of the right ventricle. A generator (Microny^®^—2525 T; St. Jude Medical, Inc) was placed under the rectus muscle, and a pacemaker rate of 100 beats per minute was programmed (Figures 2 and 5). Due to severe cardiac instability of the infant following surgery, she required a continuous infusion of dobutamine and dopamine, mechanical lung ventilation, and parenteral nutrition for the following 3 days. Following this, there was a gradual improvement with no clinical heart failure, echocardiographic evidence of ventricle dilatation, or poor systolic function. The neonate was discharged from the hospital at 9 weeks of age. At 5 years of follow‐up, the child presented with normal psychomotor development, normal heart size, and no evidence of pacemaker dysfunction (Figure 6).

## 3 DISCUSSION

Early diagnosis of isolated CCAVB is essential, allowing timely therapeutic interventions. If isolated CCAVB is suspected, it can be confirmed in utero using fetal echocardiography (the gold standard for diagnosing congenital heart block) and by using electrocardiography after birth.^1^

Congenital heart block starts to develop around the 12th week of gestation, when transplacental transfer of maternal antibodies begins,^3^ and is commonly diagnosed between 16 and 24 weeks of gestation.^1^ Correct diagnostics of isolated CCAVB depends on proper follow‐up of pregnant women with a history of a systemic disease and positive autoimmune antibodies. It is very important to recognize first signs of isolated CCAVB formation in utero, especially for women with such conditions. In the presence of positive maternal autoantibodies, serial fetal echocardiograms should be performed every 1‐2 weeks from about the 16th week of gestation in order to detect early fetal abnormalities that might precede the beginning of atrioventricular block formation.^1^, ^3^, ^11^ There are reports about effective treatment with steroids given as early as 16‐18 weeks of gestation in order to stop further atrioventricular block formation.^12^ It might be beneficial in cases with signs of myocarditis and cardiomyopathies as well. In later periods of gestation and postnatally, when complete isolated CCAVB is already formed, the treatment with steroids is controversial.

Inappropriate antenatal care of the pregnant woman and missed diagnosis of fetal complete heart block was the main reason leading to the chain of mistakes and poor outcome of the baby in our first case. The pregnant woman with a known history of a systemic disease and positive autoimmune antibodies did not get a proper follow‐up of the fetal condition; therefore, fetal CCAVB was not diagnosed and early treatment with corticosteroids and/or immunoglobulins was not started. Moreover, the delivery took place at a regional but not tertiary level hospital with the limited resources and inadequate experience of the obstetricians. Therefore, fetal bradycardia recorded on the 29th week of gestation was mistakenly assumed as a sign of acute fetal distress due to fetal hypoxia, but not due to CCAVB and an unnecessary emergency cesarean section was performed. A missed window of opportunity of early antenatal treatment with steroids as well as unnecessary preterm delivery of the baby complicated the main diagnosis of CCAVB that was diagnosed after delivery. Due to the poor baby's condition from the early beginning with the dominant signs of cardiac and respiratory insufficiency, the patient was transferred to the tertiary level hospital for further treatment and pacemaker implantation.

Isolated CCAVB typically manifests as sustained bradycardia. However, fetal sinus bradycardia can also be a sign of secondary advanced fetal hypoxia, an obstetric emergency. It is very important to distinguish CCAVB from other obstetric causes of fetal bradycardia. Mistakenly assuming fetal bradycardia as a sign of acute fetal distress due to fetal hypoxia, but not due to CCAVB can lead to unnecessary emergency cesarean section, but not to appropriate antenatal treatment of CCAVB and prolongation of pregnancy. This is especially true if a preterm birth occurs in a hospital where there is no neonatal intensive care and multidisciplinary team. Complications related to prematurity itself, together with a delayed diagnosis of isolated CCAVB, necessitate rapid transportation of the newborn to a tertiary level care center before the clinical condition worsens.

Welch et al^5^ previously reported two cases of CCAVB. In one of the cases, an emergency cesarean section was performed at 27 weeks of gestation due to a fetal heart rate of less than 90 beats per minute, without any obvious etiologies.^5^ That case report is similar to our presented case, but with a better outcome. In our first case, complete atrioventricular block and fetal bradycardia were erroneously diagnosed as acute fetal hypoxia leading to the wrong management approach—an emergency cesarean section at a regional hospital. As a result, the condition causing the fetal bradycardia—isolated CCAVB—was complicated with other life‐threatening complications related to prematurity, and ultimately a lethal outcome.

Close maternal observation during pregnancy, with careful examination of fetal well‐being and allowing term delivery is recommended if fetal isolated CCAVB is suspected. Delivery should take place at a perinatal center where high‐quality neonatal intensive care and pediatric cardiac surgery are available.

In our second case, the mother had a normal course of pregnancy without any suspicion of fetal distress until 26 weeks of gestation when obvious fetal bradycardia was detected, and isolated CCAVB was diagnosed during one of her antenatal visits. Emergency hospitalization to a tertiary level perinatal center was recommended, and autoimmune antibodies (anti‐SS‐A, anti‐Ro 52, and ANA) were identified in the blood of the pregnant women confirming the origin of isolated CCAVB. A short course of steroids was considered just for fetal lung maturation, but not for the treatment of CCAVB. Due to severe preeclampsia, an emergency cesarean section was performed at 28 weeks of gestation and a very low birth weight infant received appropriate and complex care from the first hours of life including pacemaker implantation, thus preventing the development of heart failure.

Selection of the best pacing strategy for the premature neonate remains a challenge and will depend on the practical skills of the surgeon, the surgical technique, and available devices. Severe prematurity and very low birth weight are significant risk factors for poor neonatal outcomes. The decision of implantation of a pacemaker in both of our cases was taken after discussions among neonatologists, pediatric cardiologists, and cardiac surgeons with regard to our clinical conditions and technical possibilities. Unfortunately, we did not have a clear protocol or recommendations at the moment of decision making. There is still ongoing discussion about what initial treatment option would be the best for this population of patients. Some authors suggest temporary epicardial pacing as a bridge to permanent pacemaker implantation until the clinical condition becomes stable and the neonate reaches a weight of least 2 kg.^4^, ^6^ Alternatively, a number of publications have described successful immediate permanent epicardial pacemaker implantation in low birth weight infants. Shepard and colleagues reported data from a multi‐institutional registry, which supported initial permanent epicardial pacemaker implantation in low birth weight preterm neonates with isolated CCAVB.^7^ Other authors support this approach.^5^, ^8^ However, even now there is no consensus as to which approach is better for the very low birth weight infant. Some authors have described their experience with successful pacemaker implantation in newborns weighing <1 kg. These infants underwent temporary epicardial pacing prior to receiving a permanent pacemaker at 2‐3 months of life when the neonate was in a stable condition and of an appropriate weight.^9^, ^10^

From our perspective, as well as other authors,^13^ the pacemaker rate is an important issue. An initial pacemaker rate of 130 beats per minute was programmed in our first patient. We speculate now that this programmed rate was too fast and could be one of the reasons that the infant developed congestive heart failure. As a result of our first experience, we used a lower pacemaker rate of 100 beats per minute in the second infant who had a better outcome.

Low ventricular rate, hydrops, prematurity, and dilated cardiomyopathy are mentioned in the literature among factors associated with neonatal deaths in isolated CCAVB. All of these factors were observed in case 1. An elevated concentration of proinflammatory mediators (C‐reactive protein, procalcitonin) was assumed as signs of early suspected sepsis, but also could be the markers of perinatal myocarditis due to autoimmune processes that finally led to the development of the dilated cardiomyopathy.

There are several reports that long‐term right ventricular pacing is a risk factor for left ventricular dyssynchrony, remodeling, and dysfunction.^14^, ^15^ A randomized multicenter study emphasized that different pacing sites may have a different impact on left ventricular function.^14^ That study showed that pacing the left ventricular apex, or free wall, resulted in promising outcomes conserving left ventricular synchrony and function. A prospective study from a single center supported the safety and effectiveness of the left ventricle apex or free wall pacing during short and medium‐term follow‐up.^15^ Another report described a successful case of implantation of epicardial leads on the left ventricular wall for the low birth weight preterm newborn with isolated CCAVB.^11^ In both of our patients, initial permanent epicardial pacing was initiated from the right ventricle. However, our first case electrocardiogram after pacemaker implantation revealed left bundle branch block morphology and rightward QRS axis (Figure 2), which may indicate pacing site at the right ventricular outflow tract results in higher risk of heart failure progression.^14^ Follow‐up of our surviving patient with a permanent right ventricle pacing at 5 years of age showed normal heart function with no ventricular dilatation, no left ventricular dysfunction, and no pacemaker dysfunction.

However, we still lack guidelines for best practice when dealing with isolated CCAVB in very low birth weight neonates.

## 4 CONCLUSION

Isolated CCAVB due to damage of fetal and neonatal cardiac conduction pathways is a serious, life‐threatening condition. Timely accurate diagnosis and appropriate management before and after birth are essential. Neonates with isolated congenital complete heart block should be managed in a tertiary referral center with advanced perinatal and cardiac resources.

## CONFLICT OF INTEREST

None declared.

## AUTHOR CONTRIBUTIONS

RG: involved in conception and design, drafting, and final approval of submitted version. RV: involved in conception and design, revising the article, editing, and final approval of submitted version. OK: involved in final approval of submitted version of the article. AL: involved in conception and design, revising and editing the article, and final approval of submitted version.

## ETHICAL APPROVAL

The manuscript is written with informed consent and parental permission. All the authors read and approved the final manuscript.

## References

[1] Bordachar P , Zachary W , Ploux S , Labrousse L , Haisseguerre M , Thambo J . Pathophysiology, clinical course, and management of congenital complete atrioventricular block. Heart Rhythm. 2013;10:760‐766. 23276818 10.1016/j.hrthm.2012.12.030

[2] Lee N , Shin S , Park J , Park J , Kim C , Choi H . Temporary transcutaneous pacing in a low birth weight preterm neonate with congenital complete atrioventricular block: a case report. Neonatal Med. 2016;23:223‐227.

[3] Yildirim A , Tunaoolu F , Karaaoac A . Neonatal congenital heart block. Indian Pediatr. 2013;50:483‐488. 23778728 10.1007/s13312-013-0156-3

[4] Glatz AC , Gaynor JW , Rhodes LA , et al. Outcome of high‐risk neonates with congenital complete heart block paced in the first 24 hours after birth. J Thorac Cardiovasc Surg. 2008;136:767‐773. 18805283 10.1016/j.jtcvs.2008.04.019

[5] Welch EM , Hannan RL , DeCampli WM , et al. Urgent permanent pacemaker implantation in critically ill preterm infants. Ann Thorac Surg. 2010;90:274‐276. 20609793 10.1016/j.athoracsur.2009.11.022

[6] Beake M , Bhole V , Johnston T , Rasiah S . Successful emergency cardiac pacing and permanent pacemaker insertion in a preterm 29‐week gestation hydropic baby with congenital complete heart block. Cardiol Young. 2015;25:348‐349. 24984226 10.1017/S1047951114000481

[7] Shepard CW , Kochilas L , Vinocur JM , et al. Surgical placement of permanent epicardial pacing systems in very low‐birth weight premature neonates: a review of data from the Pediatric Cardiac Care Consortium (PCCC). World J Pediatr Congenit Heart Surg. 2012;3:454‐458. 23804908 10.1177/2150135112453178

[8] Donofrio M , Gullquist S , Mehta I , Moskowitz W . Congenital complete heart block: fetal management protocol, review of the literature, and report of the smallest successful pacemaker implantation. J Perinatol. 2004;24:112‐117. 14762451 10.1038/sj.jp.7211038

[9] Filippi L , Vangi V , Murzi B , Moschetti R , Colella A . Temporary epicardial pacing in an extremely low‐birth‐weight infant with congenital atrioventricular block. Congenit Heart Dis. 2007;2:199‐202. 18377466 10.1111/j.1747-0803.2007.00098.x

[10] Nakanishi K , Takahashi K , Kawasaki S , Fukunaga H , Amano A . Management of congenital complete heart block in a low‐birth‐weight infant. J Card Surg. 2016;31:645‐647. 27573261 10.1111/jocs.12824

[11] Di Coste A , Cassano V , Troise D , Annecchino F . Pacemaker implantation in a premature low weight newborn with critical congenital atrioventricular block. G Chir. 2011;32:307‐309. 21771397

[12] Satomi G . Guidelines for fetal echocardiography. Pediatr Int. 2015;57:1‐21. 25711252 10.1111/ped.12467

[13] Chen C‐A , Chang C‐I , Wang J‐K , et al. Restoration of cardiac function by setting the ventricular pacing at a lower range in an infant with congenital complete atrioventricular block and dilated cardiomyopathy. Int J Cardiol. 2008;131:e38‐e40. 17931722 10.1016/j.ijcard.2007.07.061

[14] Janoušek J , van Geldorp IE , Krupičková S , et al. Permanent cardiac pacing in children: choosing the optimal pacing site: a multicenter study. Circulation. 2013;127:613‐623. 23275383 10.1161/CIRCULATIONAHA.112.115428

[15] Silvetti MS , Di Carlo D , Ammirati A , et al. Left ventricular pacing in neonates and infants with isolated congenital complete or advanced atrioventricular block: short‐ and medium‐term outcome. Europace. 2015;17:603‐610. 25115169 10.1093/europace/euu180

